# Targeting mTOR to Overcome Epidermal Growth Factor Receptor Tyrosine Kinase Inhibitor Resistance in Non-Small Cell Lung Cancer Cells

**DOI:** 10.1371/journal.pone.0069104

**Published:** 2013-07-16

**Authors:** Shi-Jiang Fei, Xu-Chao Zhang, Song Dong, Hua Cheng, Yi-Fang Zhang, Ling Huang, Hai-Yu Zhou, Zhi Xie, Zhi-Hong Chen, Yi-Long Wu

**Affiliations:** 1 Guangdong Lung Cancer Institute, Medical Research Center of Guangdong General Hospital and Guangdong Academy of Medical Sciences, Guangzhou, China; 2 Graduate School of Southern Medical University, Guangzhou, China; 3 Thoracic oncology, the Fifth Affiliated Hospital of Sun Yat-Sen University, Zhuhai, Guangdong, China; Indiana University School of Medicine, United States of America

## Abstract

**Aims:**

Epidermal growth factor receptor (EGFR) tyrosine kinase inhibitors (TKIs) have shown dramatic clinical benefits in advanced non-small cell lung cancer (NSCLC); however, resistance remains a serious problem in clinical practice. The present study analyzed mTOR-associated signaling-pathway differences between the EGFR TKI-sensitive and -resistant NSCLC cell lines and investigated the feasibility of targeting mTOR with specific mTOR inhibitor in EGFR TKI resistant NSCLC cells.

**Methods:**

We selected four different types of EGFR TKI-sensitive and -resistant NSCLC cells: PC9, PC9GR, H1650 and H1975 cells as models to detect mTOR-associated signaling-pathway differences by western blot and Immunoprecipitation and evaluated the antiproliferative effect and cell cycle arrest of ku-0063794 by MTT method and flow cytometry.

**Results:**

In the present study, we observed that mTORC2-associated Akt ser473-FOXO1 signaling pathway in a basal state was highly activated in resistant cells. *In vitro* mTORC1 and mTORC2 kinase activities assays showed that EGFR TKI-resistant NSCLC cell lines had higher mTORC2 kinase activity, whereas sensitive cells had higher mTORC1 kinase activity in the basal state. The ATP-competitive mTOR inhibitor ku-0063794 showed dramatic antiproliferative effects and G1-cell cycle arrest in both sensitive and resistant cells. Ku-0063794 at the IC_50_ concentration effectively inhibited both mTOR and p70S6K phosphorylation levels; the latter is an mTORC1 substrate and did not upregulate Akt ser473 phosphorylation which would be induced by rapamycin and resulted in partial inhibition of FOXO1 phosphorylation. We also observed that EGFR TKI-sensitive and -resistant clinical NSCLC tumor specimens had higher total and phosphorylated p70S6K expression levels.

**Conclusion:**

Our results indicate mTORC2-associated signaling-pathway was hyperactivated in EGFR TKI-resistant cells and targeting mTOR with specific mTOR inhibitors is likely a good strategy for patients with EGFR mutant NSCLC who develop EGFR TKI resistance; the potential specific roles of mTORC2 in EGFR TKI-resistant NSCLC cells were still unknown and should be further investigated.

## Introduction

The epidermal growth factor receptor (EGFR) signaling pathway plays a central role in the development and progression of lung cancer [1]. EGFR tyrosine kinase inhibitors (TKIs) gefitinib and erlotinib are effective clinical therapies for patients with advanced NSCLC who have EGFR-activated mutations, compared with standard first-line cytotoxic chemotherapy [2]–[4]. However, despite these dramatic benefits of EGFR TKIs, all of these patients inevitably develop resistance to gefitinib and erlotinib, usually 6–12 months after initiation of TKI treatment [5]. Several mechanisms, including a T790M mutation in the EGFR, MET amplification, and overexpression of hepatocyte growth factor (HGF), induce acquired resistance to reversible EGFR-TKIs for NSCLC with EGFR-activating mutations [6]–[8]. A means of overcoming TKI resistance remains a challenge in clinical practice. Generally, strategies to overcome resistance consider the resistance mechanism itself [7], [9], [10], whereas an alternative strategy is to identify new molecules or mechanisms that overcome the resistance, such as mTOR.

mTOR is a conserved serine/threonine kinase that occurs in mTORC1 and mTORC2 complexes [11]. It integrates signals from growth factors, nutrient supply, and energy status to activate cell growth, and is upregulated in various cancers [12]. Therefore, studies targeting mTOR for cancer therapy have received attention in recent years. However, the clinical response to rapamycin and its analogues has been feeble [13]. Many studies have demonstrated the mechanisms of its poor response both *in vitro* and *in vivo* [14]–[16]. Our previous report also showed that all NSCLC cell lines in our lab are resistant to RAD001 (a rapamycin analogue) [17]. In fact, rapamycin only partially inhibits mTORC1 functions and instead induces Akt ser473 phosphorylation [16] and does not inhibit mTORC2 at all [18]. More recent studies have indicated that mTORC2 regulates cell survival and cytoskeletal organization by regulating phosphorylation of its substrates, Akt hydrophobic motif (HM) site (ser473) and PKCα HM site (ser657) [19], [20]. mTORC2 kinase activity increases in some tumors; thus, targeting mTORC2 could inhibit cancer cell growth and proliferation [21]–[23]. Therefore, we hypothesized that mTORC2 plays an important role in cell growth and proliferation and that targeting mTOR with specific mTOR inhibitors would produce better anti-tumor effects after TKI-acquired resistance.

We first analyzed differences in the mTOR-associated signaling pathways between EGFR TKI-sensitive and -resistant NSCLC cells in a basal state *in vitro*. Then, we assayed mTORC1 and mTORC2 kinase activities *in vitro*. Finally, we evaluated the antiproliferative effects and cell cycle arrest of ku-0063794 and analyzed the expression of total and phosphorylated p70S6K in tumor specimens.

## Materials and Methods

### Antibodies and Regents

Ku-0063794 (TOCRIS, Minneapolis, MN, USA) and pure gefitinib, kindly provided by AstraZeneca, were prepared in DMSO and diluted with culture medium before use. mTOR (#2983), p-mTOR (#2971), Rictor (#2114 and #5379), Raptor (#2280 and #5382), Akt (#9272), p-Akt ser473 (#4060), p-p70S6K (#9208), p-4EBP1 (#9459), FOXO1 (#2880) and p-FOXO1 (#9461) antibodies for western blot and immunoprecipitation were all purchased from CST (Beverly, MA, USA). p70S6K (#ab47504) and p-p70S6K (#ab60947) antibodies for western blot and immunohistochemistry were purchased from Abcam (Cambridge, MA, USA). Inactive Akt1/PKB1 (#14–279) and 4E-BP1 (#10022-H07E) were purchased from Millipore (Bedford, MA, USA) and Sino Biological (Beijing, China), respectively.

### Cell Lines

The human lung adenocarcinoma cell lines PC9, H1650, and H1975 (ATCC, Manassas, VA, USA) were kindly provided by Dr. Tony Mok, Chinese University of Hong Kong. PC9 cells harbor an EGFR exon 19-frame deletion and are highly sensitive to EGFR TKI. H1650 cells harbor an EGFR exon 19-frame deletion and are resistant to EGFR TKI. H1975 cells carry the EGFR L858R-T790M mutation and are resistant to EGFR TKI. PC9GR acquired gefitinib resistance by chronic exposure of PC9 cells to medium with increasing gefitinib concentrations. Briefly, PC9 cells were exposed to 10 nM gefitinib in medium containing 10% FBS, and the concentration was increased in a stepwise manner. Cells that were able to grow in 1 µM gefitinib were obtained 6 months after the initial exposure.

### Western Blot Analysis

Cells were cultured in 25 cm^2^ culture bottles without any drugs and harvested in the log-growth phase for protein extraction. Cells were lysed in RIPA lysis buffer containing 1× PMSF and 1× phosphatase inhibitor cocktail. The protein concentration of each lysate was determined using the bicinchoninic acid assay. Samples were subjected to SDS-PAGE. Proteins were detected using the enhanced chemiluminescence kit (Thermo, Rockford, IL, USA).

For quantification of protein levels, films were scanned and analyzed with labwork software. The relative protein levels were counted using a comparison to PC9 cell.

### Immunoprecipitation and Kinase Assays

Cells in the log-growth phase were lysed in 0.5 ml of ice-cold lysis buffer (40 mM Hepes pH 7.5, 120 mM NaCl, 1 mM EDTA, 1× phosphatase inhibitor cocktail, 1× EDTA-free protease inhibitor cocktail containing 0.3% [w/v] CHAPS) [24]. The supernatant was transferred to a new tube. A 10 µl aliquot of immobilized bead conjugate with the indicated antibody was added and incubated with rotation for 90 min at room temperature. Immunoprecipitates were washed four times with CHAPS lysis buffer and once with the Rictor-mTOR kinase buffer (25 mM Hepes pH 7.5, 100 mM potassium acetate, 1 mM MgCl_2_). Immunoprecipitates were incubated in a final volume of 15 µl for 20 min at 37°C in Rictor-mTOR kinase buffer containing 500 ng inactive Akt1/PKB1 or 4E-BP1 in the presence of ATP for the kinase reaction. The reaction was stopped by addition of 200 µl ice-cold enzyme dilution buffer (20 mM MOPS, pH 7.0, 1 mM EDTA, 0.01% [w/v] Brij 35, 5% glycerol, 0.1% 2-mercaptoethanol, and 1 mg/ml BSA). After a short centrifugation, the supernatant was removed from the Sepharose bead conjugate and analyzed by immunoblotting. The immunoprecipitates were denatured and resolved by SDS-PAGE, and the gels were stained with a silver staining kit.

### Evaluation of Antiproliferative Effects

Cell viability was determined via the MTT assay, in accordance with the method of Mosmann and Carmichael [25], [26], and linearity between cell numbers and optical density values was established. The antiproliferative activity of single-agent treatment was assessed in single monolayers, with cells grown in 96-well plates. The number of cells per well was 2,000 PC9, 2,000 PC9GR, 1,200 H1650, and 3,000 H1975 cells. The IC_50_ value was the concentration resulting in 50% inhibition of cell growth following a 72-hr exposure to the drug compared with that in untreated control cells.

### Flow Cytometric Analysis of Cell Cycle Distribution

Cells were seeded into six-well plates at a density of 1×10^5^ per well and left to settle overnight. Then cells were treated for 72 hr with ku-0063794 at IC_50_ concentration. The cells were trypsinized, counted, washed, and resuspended. Cells were then pelleted and fixed by dropwise addition of 70% ice-cold ethanol, stored in PBS, and then resuspended in propidium iodide solution (180 ug/ml) and analyzed using flow cytometry.

### Patients with NSCLC

Tumor specimens from gefitinib or erlotinib-treated patients were obtained from Guangdong General Hospital (Guangzhou, China). The study was approved by the ethics committee of Guangdong General Hospital. All patients provided written informed consent. The presence of the EGFR mutation in each specimen was confirmed by exon-specific amplification (exons 18–21), followed by direct sequencing or the Scorpion amplification refractory mutation system [27]. MET amplification was analyzed by quantitative relative real-time polymerase chain reaction [28].

### p70S6K Immunohistochemistry

Immunohistochemistry for total and phosphorylated p70S6K was performed on positively charged glass slides containing 5-µm sections of formalin-fixed, paraffin-embedded tissue. Slides were deparaffinized, followed by rehydration with serially decreasing ethanol concentrations, and then immersed in 3% H_2_O_2_ to inhibit endogenous peroxidase activity. Following antigen retrieval in 1 mM EDTA buffer, pH 8.0, the tissue sections were incubated with blocking buffer (TBST/5% normal goat serum) for 1 hr at room temperature. The sections were then incubated overnight at 4°C with primary antibody diluted 1∶100 in antibody diluent. The Envision Detection System was used for visualization, according to the manufacturer’s instructions. Sections were counterstained with Harris hematoxylin, dehydrated, and mounted permanently.

### Evaluation of Immunohistochemical Staining

All slides were evaluated independently by two pathologists who were blinded to the cases. Cases with staining in >10% of cells were considered positive. Immunohistochemical reactivity was graded on a scale of 0–3 according to staining intensity and the percentage of immunopositive cells as follows: 0, no staining; <10% positive cells; 1, weak staining in >10% of tumor cells or moderate staining in 10–40% of tumor cells; 2, moderate staining in >40% of tumor cells or strong staining in 10–40% of tumor cells; 3, strong staining in >40% of tumor cells [29].

### Statistics

Results are presented as means ± standard errors of at least three experiments.

## Results

### Akt ser473-FOXO1 Signaling Pathway in EGFR TKI-resistant NSCLC Cells was Highly Activated in the Basal State

**Figure 1:**
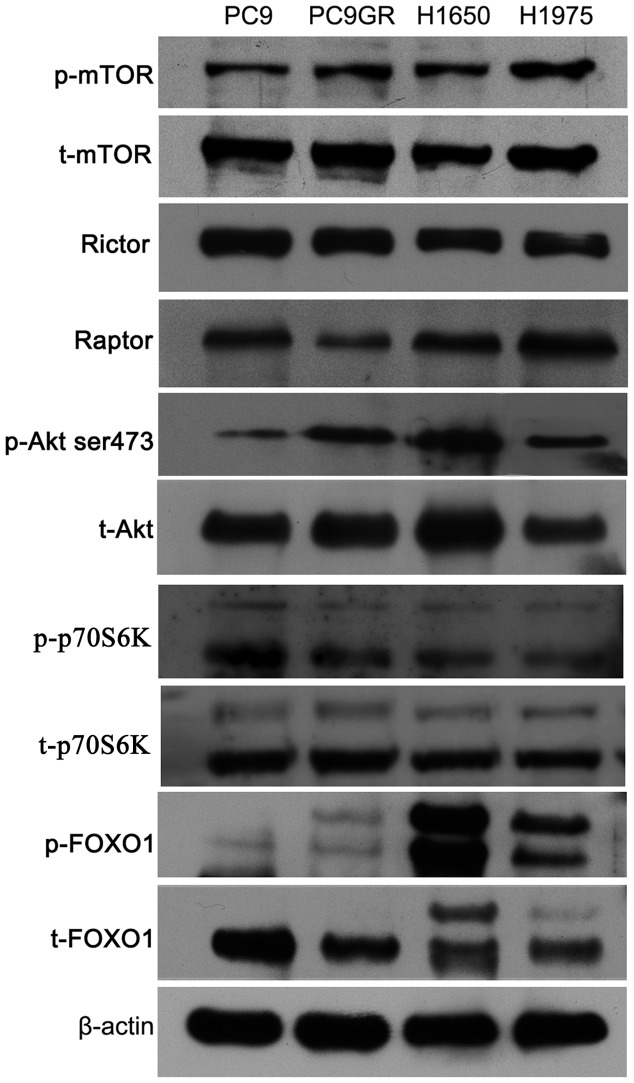
Analysis of the mTORC2-associated signaling pathway in the basal state. Cells were cultured in complete media without drug treatment. Proteins were extracted from cells in the log-growth phase, and cell lysates were immunoblotted to detect the indicated proteins. The experiment, repeated three times, yielded similar results.

Understanding mTOR-associated signaling-pathway differences between EGFR TKI-sensitive and -resistant NSCLC cells is critical for targeting mTOR to overcome patient resistance after TKI treatment. However, knowledge of the differences in signaling pathways between EGFR TKI-sensitive and -resistant NSCLC cells is lacking. Here, we first selected four different NSCLC cell lines that harbor different EGFR mutants, and that differ in sensitivity to TKIs, as cell models for analysis of the signaling-pathway differences in a basal state by western blot. As shown in Fig. 1, the total and phosphorylated mTOR, Raptor, and Rictor proteins had higher basal expression levels in all four NSCLC cell lines. Then, we detected differences in the mTORC2-associated signaling pathway by determining the phosphorylation status of Akt ser473 and FOXO1. Interestingly, we found that EGFR TKI-resistant cell lines especially H1650 cells (harboring the EGFR deletion in 19 exon and resistant to TKI) had higher p-Akt ser473 levels than did TKI-sensitive cells (PC9 cells) and p-Akt ser473 expression level in PC9GR cells (gefitinib-acquired resistant cell line) was also quite high. This result was consistent with a report that PC9GR cells have reduced phosphatase and tensin homolog expression, which led to upregulation of p-Akt ser473 levels [30]. FOXO1 phosphorylation status, which is positively regulated by the Akt HM site (ser473) phosphorylation status and inhibits cell apoptosis [31], was almost consistent with the p-Akt ser473 levels in the four cell lines. We also detected the expression of phosphorylated and total p70S6K which is the substrate of mTORC1 and found that all four cell lines had higher p70S6K phosphorylation levels. Taken together, our data indicated that EGFR mutant NSCLC cells had higher basal expression levels of mTOR, Rictor and Raptor and mTORC2-associated Akt ser473-FOXO1 signaling pathway was hyperactivated in resistant cells.

### EGFR TKI-resistant NSCLC Cell Lines had Higher mTORC2 Kinase Activity, whereas Sensitive Cells had Higher mTORC1 Kinase Activity in the Basal State

**Figure 2:**
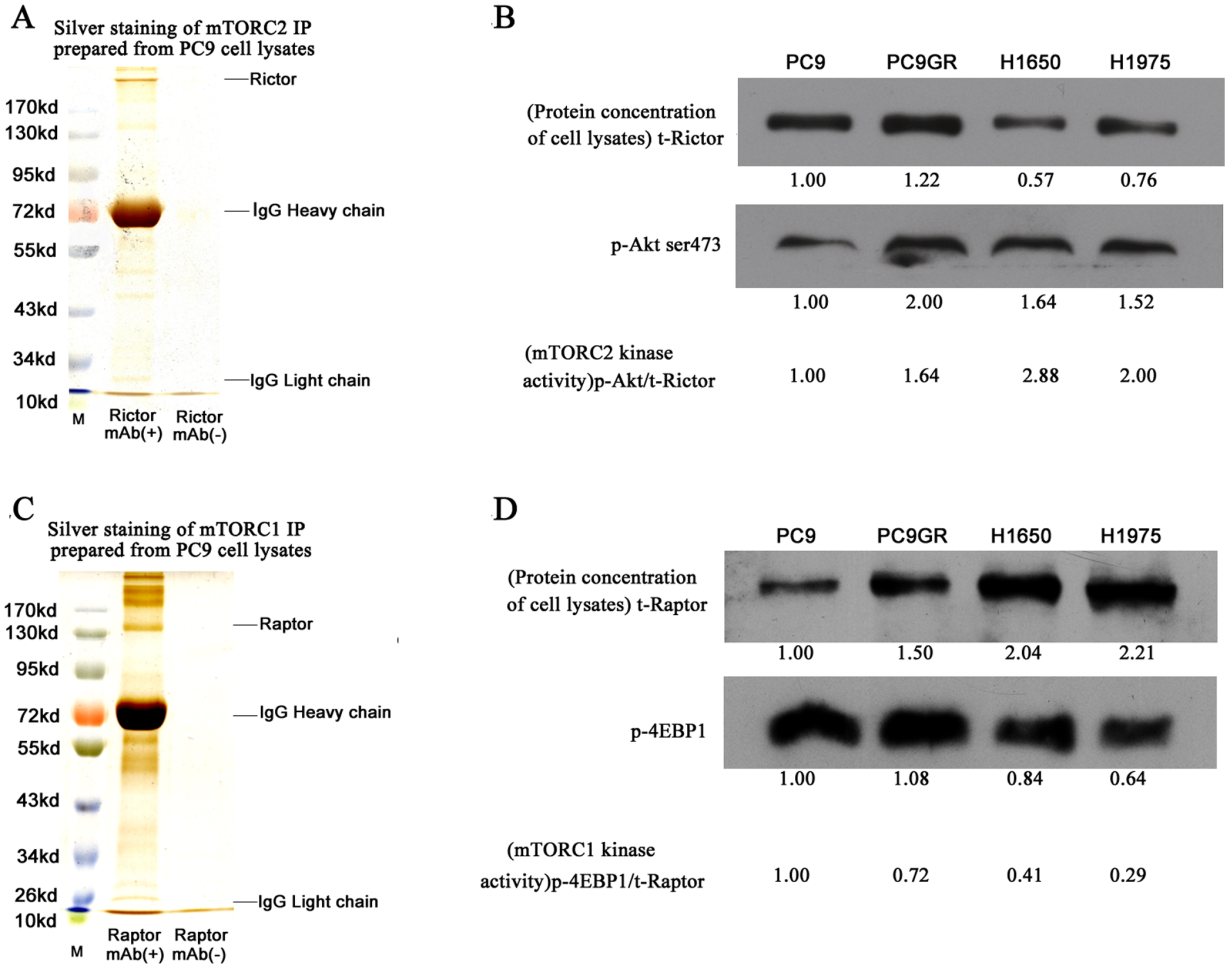
*In vitro* mTORC2 and mTORC1 kinase activity assay in the basal state. (A, C) SDS-PAGE protein silver staining of the mTORC2 and mTORC1 immunoprecipitates prepared from PC9 cell lysates with Rictor (D16H9) Rabbit mAb (Sepharose Bead Conjugate) (#5379) and Raptor (24C12) Rabbit mAb (Sepharose Bead Conjugate) (#5382) purchased from CST, respectively. (B, D) The first column represents the Rictor and Raptor protein concentrations, respectively, in cell lysates. The relative protein levels are counted using a comparison to PC9 cell which defined as 1.00. The immunoprecipitates were used in kinase assays with full-length, wild-type recombinant human Akt1/PKB1 and 4E-BP1 as substrates. Immunoblotting was used to detect Akt/PKB phosphorylation at ser 473 and 4E-BP1 at thr 37/46, respectively. The ratio values of p-Akt ser473/t-Rictor and p-4EBP1/t-Raptor represent kinase activity of mTORC2 and mTORC1 respectively. The experiment, repeated three times, yielded similar results.

Based on the above results, we hypothesized that EGFR TKI-sensitive and -resistant cells have different mTORC2 kinase activity. We then assayed the mTORC2 kinase activity in the four NSCLC cell lines by immunoprecipitation. First, we used the specific Rictor antibody to pull down mTORC2 into the immunoprecipitate. SDS-PAGE silver staining (Fig. 2A) showed that the immunoprecipitations were successful which were also verified by western blot of the immunoprecipitates (Fig. S1). Akt ser473 was one of the identified substrates of mTORC2 and several papers used it as an surrogate to represent mTORC2 activity [21], [24]. So, we used the inactive recombinant protein, Akt1/PKBα, as a substrate to react with the immunoprecipitate *in vitro*, and then detected the p-Akt ser473. As shown in Fig. 2B, although cell lysate protein concentration in the H1650 cell immunoprecipitate was lower than that of the other three cell lines, mTORC2 kinase activity was the highest. From Fig. 2B, we could also see that mTORC2 kinase activity in PC9 cells was the lowest, and that in PC9GR and H1975 cells had intermediate kinase activity. These results were almost consistent with the p-Akt ser473 levels mentioned above. We also detected mTORC1 kinase activity *in vitro* to compare the differences between mTORC2 and mTORC1 kinase activities in EGFR TKI-sensitive and resistant NSCLC cells. Fig. 2C showed that we also successfully pulled down mTORC1. As shown in Fig. 2D, although the protein concentration in PC9 cell immunoprecipitate was lower than that in the other three cells, mTORC1 kinase activity was the highest. mTORC1 kinase activity was lowest in H1650 and H1975 cells. From the Fig. 2B and 2D, we could also see that in the same cells when mTORC2 kinase activity was upregulated the mTORC1 kinase activity would be downregulated indicating that whether mTORC1 and mTORC2 exist in dynamic equilibrium. Taken together, our results showed that although both EGFR TKI-sensitive and -resistant NSCLC cells had higher mTORC1 and mTORC2 expression in the basal state, EGFR TKI-resistant cells had higher mTORC2 kinase activity, whereas EGFR TKI-sensitive cells had higher mTORC1 kinase activity.

### Ku-0063794 Inhibited Cell Proliferation and Resulted in G1 Cell Cycle Arrest in EGFR TKI-sensitive and -resistant NSCLC Cells

**Figure 3:**
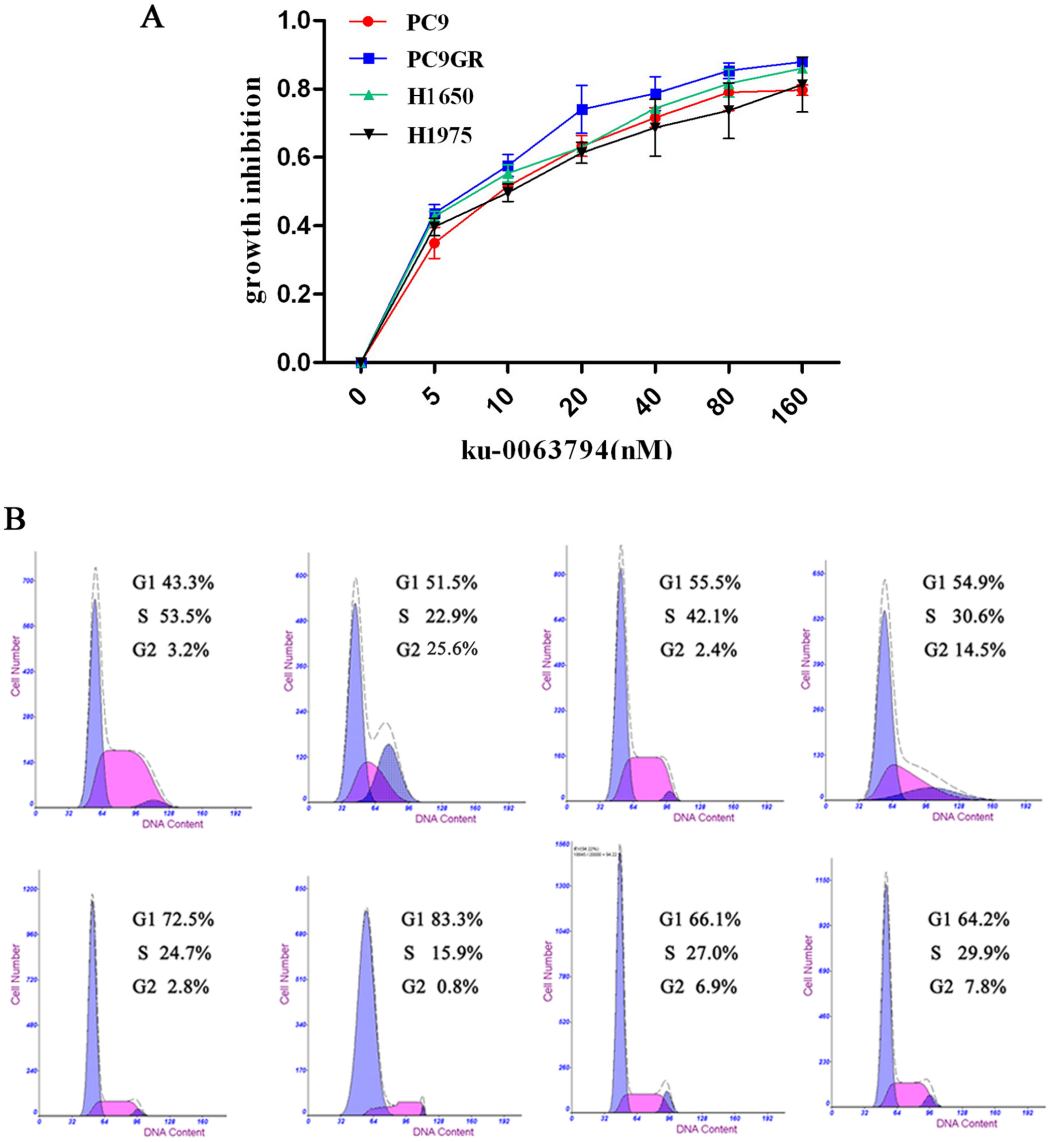
Ku-0063794 inhibited cell proliferation and induced G1 cell cycle arrest in EGFR mutant NSCLC cells. (A) Inhibition efficiency of ku-0063794 in the four NSCLC cell lines. (B) Ku-0063794 induced cell cycle arrest in both EGFR TKI-sensitive and -resistant cells. The upper panel represents basal cell cycle distributions of the PC9, PC9GR, H1650, and H1975 cell lines, respectively, and the lower panel represents the cell cycle distributions of PC9, PC9GR, H1650, and H1975 cells after treatment with the IC_50_ concentrations of ku-0063794 for 72 hr.

Selective mTOR inhibitors, such as ku-0063794, inhibit both mTORC1 and mTORC2 in different cell lines [32]. In this study, we assessed the antiproliferative effects of ku-0063794 in EGFR TKI-sensitive and -resistant NSCLC cells compared to those of gefitinib which were previously reported in our lab [17], [33]. Data indicated dose-response growth inhibition effects in PC9, PC9GR, H1650, and H1975 cells. Table 1 and Fig. 3A showed that ku-0063794 inhibited cell proliferation in both EGFR TKI-sensitive and -resistant NSCLC cells at nanomolar (nM) concentrations, whereas gefitinib inhibited only PC9 cells at nM concentrations. Greater gefitinib concentrations (µM) were needed to reach the IC_50_ value in EGFR TKI-resistant cells, which greatly exceed the peak plasma concentrations in patients [34]. We also assessed the cell cycle after treatment with ku-0063794 at IC_50_ level by flow cytometry and found that all four cell lines were blocked in the G1 phase after a 72-hr ku-0063794 treatment, particularly PC9 and PC9GR cells, compared with that in control cells without any drug treatment (Fig. 3B).

**Table 1:** IC_50_ values for each drug were calculated by performing dose response experiments with ku-0063794 and gefitinib.

IC_50_	PC9	PC9GR	H1650	H1975
Ku-0063794	10.15±0.62 nM	6.21±1.30 nM	7.61±0.62 nM	11.15±0.93 nM
Gefitinib	16.53±2.68 nM	3.01±0.36 uM	14.63±0.56 uM	6.37±1.68 uM

Each test was repeated in triplicate.

**Figure 4:**
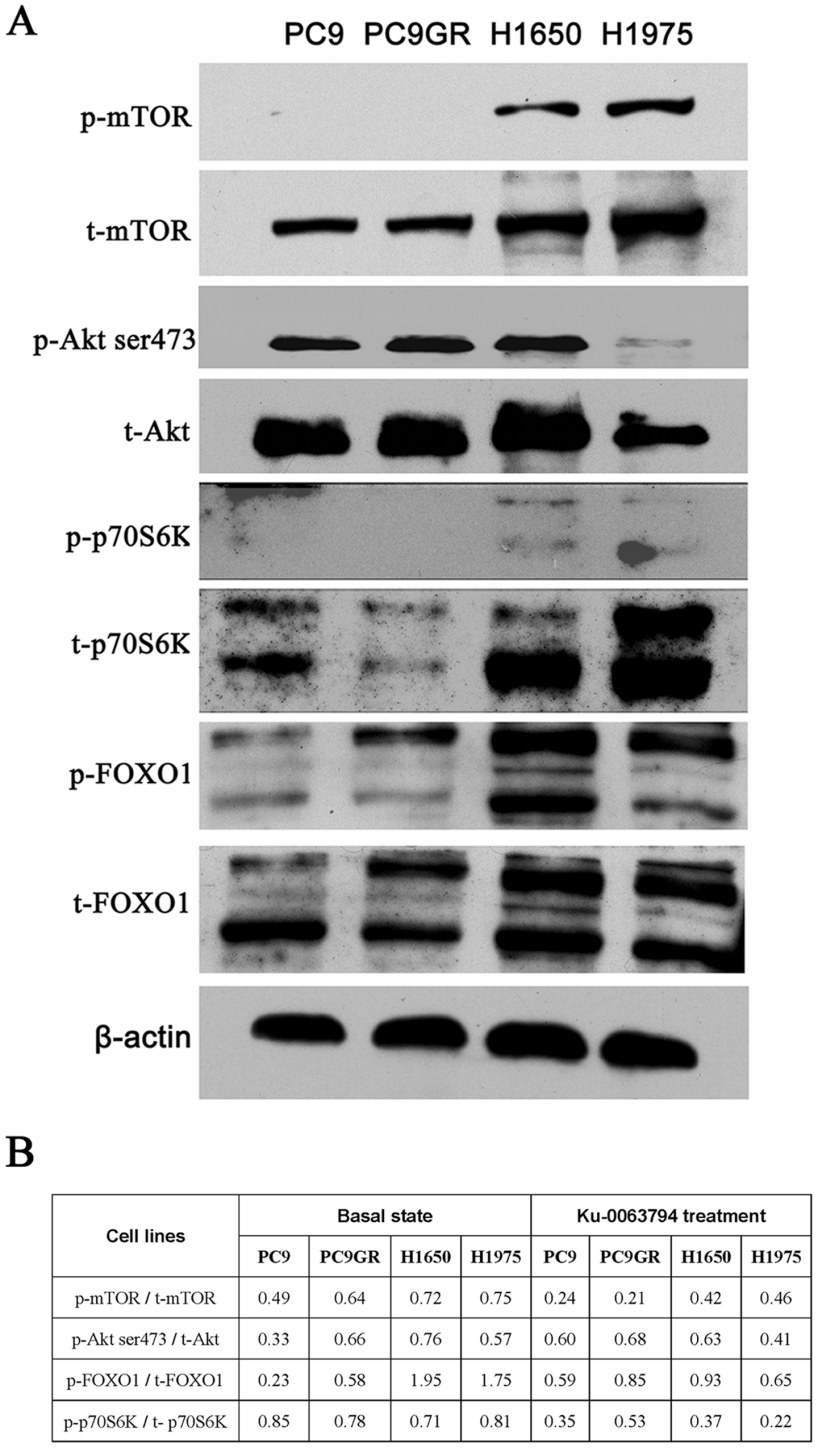
Antiproliferative effects of ku-0063794 on EGFR TKI-sensitive and -resistant NSCLC cells. (A) Cells were treated for 72 hr with ku-0063794 at the IC_50_ concentrations, and cell lysates were immunoblotted to detect the indicated proteins. The experiment, repeated three times, yielded similar results. (B) The ratios of mTOR signaling pathway associated phosphorylated proteins over total proteins in the basal state and after treatment with ku-0063794 at the IC_50_ concentrations in all four cell lines.

Because ku-0063794 exhibited dramatic antitumor effects, we further analyzed its antiproliferative activity at the IC_50_ concentration by western blot. First, we detected mTOR phosphorylation status after treatment with ku-0063794 at the IC_50_ concentrations in all four cell lines, respectively (Fig. 4A). Then we further analyzed the phosphorylation status of mTOR signaling pathway associated proteins (Fig. 4A). Through the protein quantitative labwork software, we analyzed the ratio of phosphorylated protein over total protein in the basal state and after treatment with ku-0063794 at the IC_50_ concentrations in all four cell lines (Fig. 4B). As shown in Fig. 4A and 4B, ku-0063794 effectively inhibited mTOR phosphorylation status and then strongly inhibited phosphorylation of p70S6K which is a substrate of mTORC1. From Fig. 4B, we could also see that ku-0063794 at the IC_50_ concentrations did not markedly increase p-Akt ser473 expression especially in PC9 and PC9GR cells which could be induced by rapamycin [16]. Actually, the ratios of p-Akt ser473/t-Akt in H1650 and H1975 cells decreased resulting in the reduced ratios of p-FOXO1/t-FOXO1 in these cells compared to that in the basal state. Thus, ku-0063794, as a novel ATP-competitive mTOR inhibitor, showed marked antiproliferative effects and induced G1 cell cycle arrest both in EGFR TKI-sensitive and -resistant NSCLC cells *in vitro*.

### Analyses of Clinical EGFR TKI-sensitive and -resistant Lung Adenocarcinoma Tissues Showed Higher Total and Phosphorylated p70S6K Expression in Both Sensitive and Resistant Cancers

**Figure 5:**
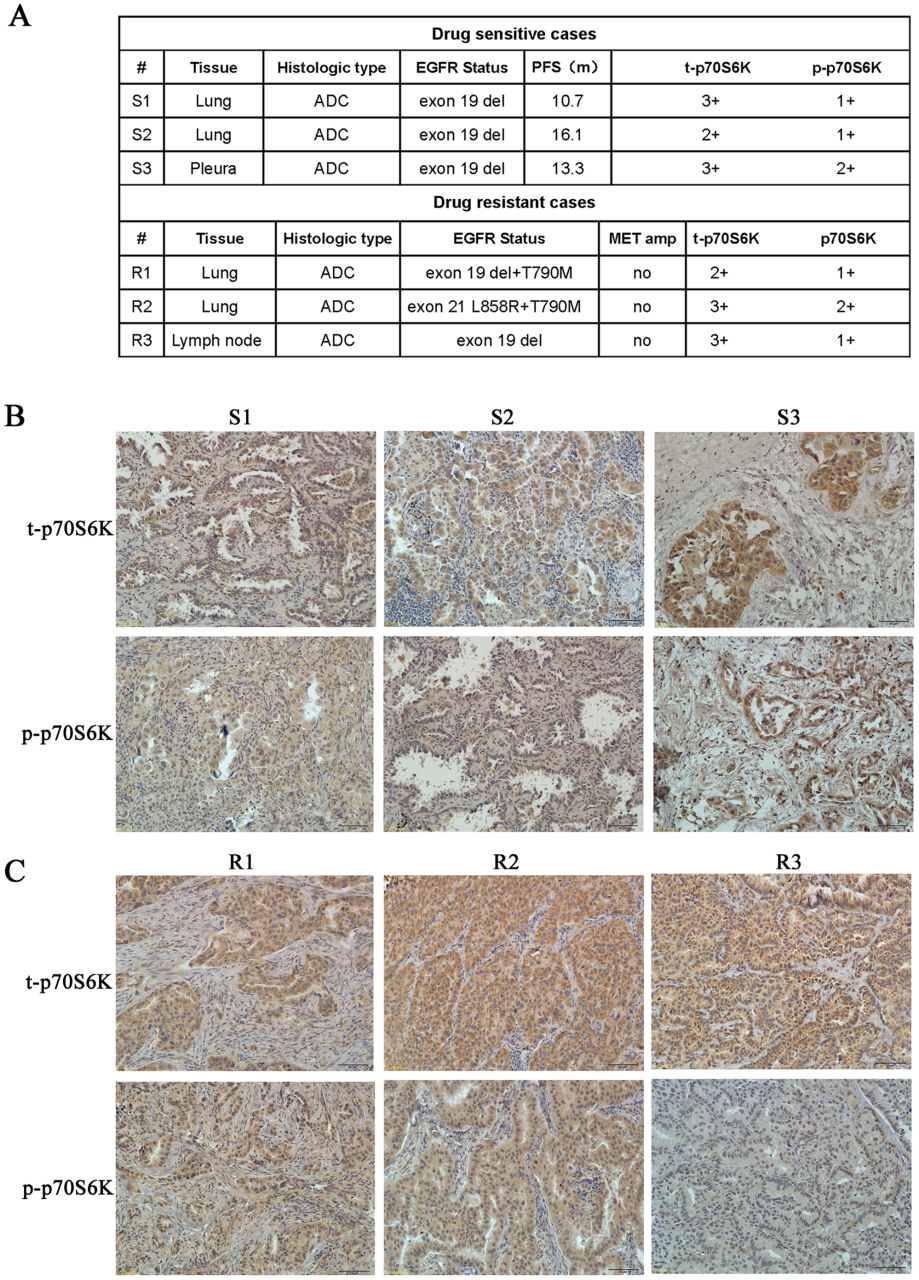
Expressions of total and phosphorylated p70S6K in clinical TKI-sensitive and -resistant lung adenocarcinoma tumor tissues. (A) Summary of the tumors in gefitinib and erlotinib-treated patients, including three drug-sensitive and -resistant cases, respectively. Case 3 was a paired sample before and after TKI treatment. ADC represents adenocarcinoma. (B) Expression of total and phosphorylated p70S6K in the EGFR TKI-sensitive cases. The upper panel represents total p70S6K, and the lower panel represents phosphorylated p70S6K. (C) Expression of total and phosphorylated p70S6K in the EGFR TKI-resistant cases. The upper panel represents total p70S6K, and the lower panel represents phosphorylated p70S6K. ADC represents adenocarcinoma; S and R represent sensitive case and resistant case respectively. Scale bars, 50 µm.

Since ku-0063794 at the IC_50_ concentration effectively inhibited p70S6K phosphorylation levels in both sensitive and resistant NSCLC cells, these results indicated that ku-0063794 may exert greater antitumor effects in tumors that express high levels of total or phosphorylated p70S6K. We examined tumor specimens from gefitinib- or erlotinib-treated patients with EGFR-mutant NSCLC (Fig. 5). All patients had a partial clinical tumor response to gefitinib or erlotinib treatment and subsequently developed clinical drug resistance. We evaluated the expression of total and phosphorylated p70S6K in five patients by immunohistochemistry; one with paired specimens obtained before and after geftinib treatment, two with drug-sensitive specimens, and two with drug-resistant specimens. We detected the status of the T790M mutation and MET amplification in all drug resistant specimens. Both drug-sensitive and drug-resistant tumor specimens had higher total p70S6K expression (Fig. 5B and 5C). We also found higher phosphorylated p70S6K expression in these tumors. These findings suggest that both drug-sensitive and -resistant cancers had higher total and phosphorylated p70S6K expression, and that p70S6K may be a good predictor of a response to ku-0063794.

## Discussion

EGFR signaling pathways are involved in the development and progression of lung cancer. Binding of EGFR to its EGF ligand leads to dimerization and initiates a series of downstream signaling cascades through the PI3K-Akt-mTORC1, Erk, and STAT3 pathways [35]. Previous studies have demonstrated that the signaling patterns activated by EGFR mutants differ from those of ligand-activated wild-type EGFR, and that EGFR-sensitive mutants resulting from EGFR signaling constitutively activate and escape negative regulation [36]–[39]. EGFR-sensitive mutations have became the curative effect predictors for TKIs, and the dramatic clinical benefits of TKIs are largely based on a deep understanding of EGFR-sensitive mutant signaling pathways. Therefore, understanding changes in signaling pathways after patients develop resistance to EGFR TKIs is quite important to overcome resistance. Several TKI resistance mechanisms have been demonstrated in recent years, including the T790M mutant [40], MET amplification [7], and HGF overexpression [8]. Most current strategies for overcoming TKI-acquired resistance involve targeting the resistance mechanism itself. For example, the second generation of irreversible TKIs such as BIBW 2992 for patients with a T790M mutation and MET inhibitors combined with EGFR TKIs for MET amplification [7], [9]–[10]. Here, we provided a new perspective to seek new molecular targets for overcoming resistance by systematically analyzing signaling pathway differences between EGFR TKI-sensitive and -resistant NSCLC cells *in vitro*. Our data showed that mTORC2-associated Akt ser473-FOXO1 signaling pathway was highly activated in EGFR TKI-resistant NSCLC cells in the basal state and *in vitro* mTORC1 and mTORC2 kinase activities verified these results.(Fig. 1 and 2). These results indicate that targeting mTOR including mTORC2 could achieve better antitumor effects.

mTOR is a central controller of cell growth, proliferation, metabolism, and angiogenesis; however, current mTOR inhibitors, such as RAD001, show only modest clinical activity against pretreated NSCLC [14]. Indeed, rapamycin targeted only a subset of the intracellular activities of mTORC1. Rapamycin allosterically inhibits mTORC1 kinase activity by binding the FKBP12 cellular protein distal to the kinase active site [15]. In some cases, mTORC1 is sensitive to rapamycin and completely inhibits mTORC1-mediated S6K1 phosphorylation. As reported previously, rapamycin induces Akt ser473 phosphorylation which reduces its antitumor effect through Akt ser473-FOXO1 signaling pathway due to relief of feedback of S6K-IRS-PI3K signalling [16], [41]. Actually, Akt thr308 phosphorylation is positively regulated by PI3K-PDK1 and Akt ser473 phosphorylation is positively controlled by mTORC2 [20], suggesting that whether inhibition of mTORC1 would promote mTORC2 kinase activity. Our data indicate a dynamic balance between mTORC1 and mTORC2 which should be further confirmed in the next studies (Fig. 2B and 2D) and explain why RAD001 mediated effects are feeble in all NSCLC cell lines *in vitro*. The results suggest that both mTORC1 and mTORC2 should be targeted.

The novel ATP-competitive mTOR inhibitors (*e*.*g*., Torin1, PP242, ku-0063794, and WAY-600), which inhibit both mTORC1 and mTORC2, exert marked effects on protein synthesis, cell growth, and cell proliferation, and strongly promote autophagy in various cancer cell types *in vitro* [32], [42]. These mTOR inhibitors are still in the early stage of evaluation; thus, their therapeutic potential for cancer remains uncertain. Indeed, novel mTOR inhibitors, such as Torin1 and PP242, have antiproliferative effects that are mediated mainly by complete inhibition of mTORC1, but not mTORC2 [43], [44]. Our previous paper and the paper from La Monica have both shown that EGFR TKI-resistant NSCLC cells were insensitive to everolimus (RAD001), and everolimus could synergize with gefitinib which provided another alternative strategy to overcome EGFR TKI resistance [17], [45]. In our study, we found that targeting mTOR with ku-0063794 also produced better antitumor effects and reduced G1 cell cycle arrest in both sensitive and resistant cells through MTT assay and flow cytometry analysis which should be further strengthened by colony formation capacity (CFC) assays in the future; we did not find significant G1-cell cycle arrest although cell proliferation was inhibited in H1975 cells by ku-0063794 which maybe primarily due to other forms of cell death like apoptosis or autophagy. Indicators of apoptosis and/or autophagy would be further investigated in the future (Fig. 3B); the following antiproliferative effects of targeting mTOR indicated that ku-0063794 not only inhibited p70S6K phosphorylation levels but also partially inhibited FOXO1 phosphorylation levels and did not upregulate Akt ser473 phosphorylation levels (Fig. 4). Taken together, our data and those from a previous report [46] suggest that the lack of an increase in Akt ser473 phosphorylation levels may have been due to inhibition of mTORC2; thus promoting antitumor effects. Actually, the potential specific roles of mTORC2 in EGFR TKI-resistant NSCLC cells were still unknown and should be further studied in the next studies. In addition, we found that EGFR TKI-resistant cells had higher Akt ser473 phosphorylation. As reported previously, Akt inhibitor perifosine have shown better antiproliferative effects in Relapsed/Refractory Waldenstrom’s Macroglobulinemia [47] and Jeong et al also reported that Akt inhibitor and mTORC1 inhibitor synergistically increased cell death in NSCLC cells [48]. All of these suggest that Akt inhibitors may be another good alternative treatment for TKI insensitive tumors. We also evaluated the expression of total and phosphorylated p70S6K in clinical EGFR TKI-sensitive and -resistant lung adenocarcinoma tumor tissues and found that p70S6K may be a good marker for selection of patients for whom ku-0063794 therapy is appropriate.

### Conclusions

In summary, we demonstrated that EGFR mutant NSCLC cell lines had different activation of mTOR-associated signaling pathways in the basal state and mTORC2-associated Akt ser473-FOXO1 signaling pathway was highly activated in resistant cells. *In vitro* mTORC1 and mTORC2 kinase activities verified these results and showed that mTORC1 and mTORC2 maybe exist in a dynamic balance. Our study also showed that ATP-competitive mTOR inhibitor ku-0063794 had dramatic antiproliferative effects and G1-cell cycle arrest in both sensitive and resistant cells. Our results indicate mTORC2-associated signaling-pathway was hyperactivated in EGFR TKI-resistant cells and targeting mTOR with specific mTOR inhibitors is likely a good strategy for patients with EGFR mutant NSCLC who develop EGFR TKI resistance; the potential specific roles of mTORC2 in EGFR TKI-resistant NSCLC cells were still unknown and should be further investigated; and that expression of total or phosphorylated p70S6K may be a predictor of the response to mTOR inhibitors.

## Supporting Information

Figure S1 **Western blot analysis of mTORC2 immunoprecipitates.** Immunoprecipitation of NSCLC cell lysates in the basal state using Rictor (D16H9) Rabbit mAb (Sepharose Bead Conjugate). The western blot was probed using Rictor Rabbit mAb.
(TIF)
