# Supplementary figures and images for: Targeting mTOR to Overcome Epidermal Growth Factor Receptor Tyrosine Kinase Inhibitor Resistance in Non-Small Cell Lung Cancer Cells
Source: PLoS One. 2013 Jul 16;8(7):e69104. doi: 10.1371/journal.pone.0069104 (PMC3712950; doi:10.1371/journal.pone.0069104)

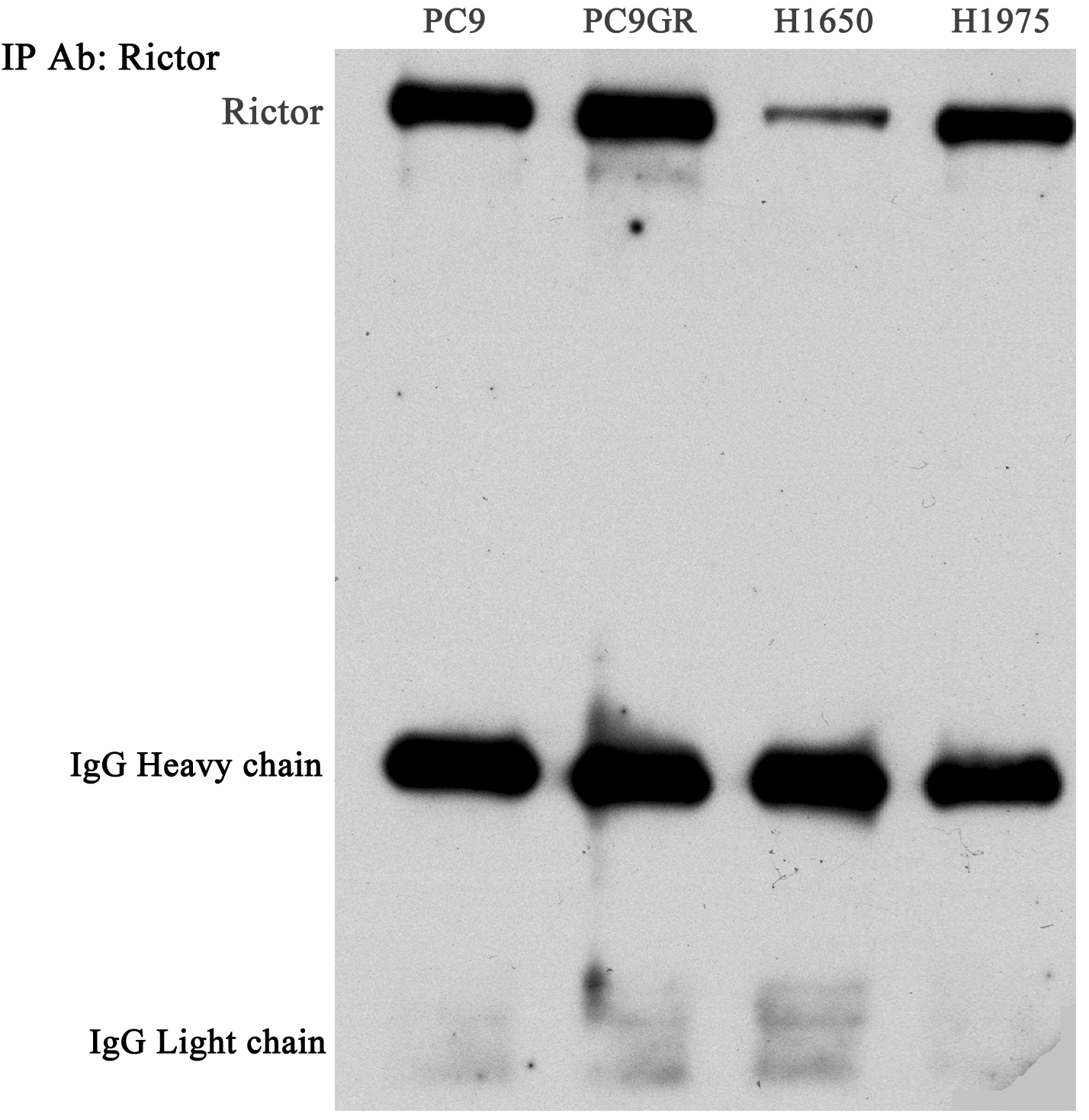

Supplement: Figure S1 — Western blot analysis of mTORC2 immunoprecipitates. Immunoprecipitation of NSCLC cell lysates in the basal state using Rictor (D16H9) Rabbit mAb (Sepharose Bead Conjugate). The western blot was probed using Rictor Rabbit mAb. (TIF) [file pone.0069104.s001.tif]
